# A serum galactomannan and neutrophil-to-lymphocyte ratio-based nomogram for predicting in-hospital mortality in non-neutropenic invasive pulmonary aspergillosis

**DOI:** 10.3389/fcimb.2025.1675277

**Published:** 2025-10-22

**Authors:** Xinyu Wang, Wenjuan Li, Yunyu Ma, Yi Li, Shengyan Ding, Jiayi Shen, Tingting Zhao, Yajie Lu, Chao Sun, Xin Su

**Affiliations:** ^1^ Department of Respiratory and Critical Care Medicine, Nanjing Drum Tower Hospital, Affiliated Hospital of Medical School, Nanjing University, Nanjing, China; ^2^ Center for Medical Big Data, Nanjing Drum Tower Hospital, Nanjing, China; ^3^ Department of Respiratory and Critical Care Medicine, Nanjing Drum Tower Hospital Clinical College of Nanjing Medical University, Nanjing, China; ^4^ Department of Respiratory and Critical Care Medicine, Nanjing Drum Tower Hospital Clinical College of Nanjing University of Chinese Medicine, Nanjing, China; ^5^ Department of Respiratory and Critical Care Medicine, Jinling Hospital, Affiliated Hospital of Medical School, Nanjing University, Nanjing, China

**Keywords:** non-neutropenic invasive pulmonary aspergillosis, NLR, serum GM, prognostic prediction, nomogram

## Abstract

**Background:**

We developed a novel prognostic model combining serum galactomannan (GM) and neutrophil-to-lymphocyte ratio (NLR) for predicting in-hospital mortality in non-neutropenic invasive pulmonary aspergillosis (IPA).

**Methods:**

We retrospectively identified 278 eligible IPA patients using the automated natural language processing system. Patients were divided into survival (n=199) and non-survival (n=79) groups. A multivariate logistic regression was developed to predict in-hospital mortality based on age, critical condition, serum GM and NLR. Internal validation was performed using bootstrap resampling methods. Subsequently, a clinical nomogram was constructed to support clinical decision-making.

**Results:**

Serum GM exhibited a higher specificity of 78.9% (95% CI: 72.6–84.3%), whereas NLR demonstrated relatively greater sensitivity at 70.9% (95% CI: 59.6–80.6%) for mortality prediction. The combination of either positive exhibited a sensitivity of 81.0%, while the dual-positive criterion achieved a specificity of 90.5%. A comprehensive model integrating age, critical condition, serum GM, and NLR demonstrated robust discrimination, with a mean AUC of 0.815 (SD = 0.005) after bootstrap resampling with 1000 iterations. This result was consistent with the original model AUC of 0.818 (95% CI: 0.767-0.870). Decision curve analysis further confirmed that the comprehensive model provided a greater net benefit compared to the simple model based solely on age and critical condition, highlighting the clinical utility of including serum GM and NLR as biomarkers in decision-making processes.

**Conclusion:**

Our study provided novel evidence that the synergistic integration of mycological (serum GM) and inflammatory biomarkers (NLR) significantly improves prognostic accuracy in non-neutropenic IPA. The developed clinical nomogram offers a practical tool to support clinical decision-making.

## 1 Introduction

Invasive pulmonary aspergillosis (IPA) is a severe disease due to its high morbidity and mortality rate (Li et al., 2021; Soontrapa et al., 2022). Approximately 2.1 million annual IPA cases occur among patients with chronic obstructive pulmonary disease (COPD), critical illness in intensive care unit (ICU), lung cancer, or hematological malignancies, with an estimated crude mortality of 85.2% (1.8 million deaths annually) (Denning, 2024). These estimates are grounded in models and literature reviews, accompanied by uncertainties, but they highlight the substantial global burden of IPA.

Clinical manifestations, imaging changes, and dynamic changes of serum galactomannan (GM) test are frequently employed indicators for diagnostic evaluation and therapeutic monitoring (Slavin et al., 2021). Nevertheless, the imaging changes often lag behind the actual pathological progression (Maertens et al., 2016). GM is a type of polysaccharide that is widely present in the cell walls of *Aspergillus* species. In the early stages of *Aspergillus* invasion, GM is shed into the bloodstream. The quantity of serum GM released is directly proportional to the fungal burden and serves as a reliable indicator of infection severity (Ergün et al., 2021). However, the sensitivity of serum GM may be compromised by antifungal treatment. Prognostic indicators that allow early risk stratification remain suboptimal. This underscores the need to complement fungal burden markers with host-related indicators.

The immune-inflammatory status of the host and the fungal load collectively determine IPA progression. While innate immunity provides the first-line defense, adaptive immunity is also critical for *Aspergillus* clearance (Sarden et al., 2022). However, excessive inflammatory responses could aggravate lung tissue damage through oxidative stress and cytokine storms (Lee et al., 2020). Notably, elevated inflammatory markers (such as C-reactive protein (CRP) and interleukin-6) correlate with poor outcomes in IPA (Tong et al., 2021; Li et al., 2023b). The neutrophil-to-lymphocyte ratio (NLR) has gained attention as a new prognostic marker in acute respiratory infections (Wang et al., 2023; Li et al., 2024). Neutrophils are essential for antifungal defense, but excessive neutrophil activation and the release of neutrophil extracellular traps can cause collateral lung damage (Praneechit et al., 2024).

Despite these insights, the prognostic value of combining inflammatory biomarkers with fungal burden indicators (serum GM) has not been systematically studied in non-neutropenic IPA. This dual perspective from both pathogen and host provides a novel framework for prognostic assessment. Therefore, this study aimed to evaluate whether integrating serum GM and NLR can predict in-hospital mortality in non-neutropenic IPA patients.

## 2 Methods

### 2.1 Study design and participants

We retrospectively identified all IPA patients from electronic medical record system of Nanjing Drum Tower Hospital from January 2018 to October 2024 using the automated natural language processing system, supported by the Center for Medical Big Data. All cases were reconfirmed by two respiratory specialists according to the guidelines of the European Organization for Research and Treatment of Cancer and the Mycoses Study Group Education and Research Consortium(EORTC/MSGERC) updated in 2020, Invasive Fungal Diseases in Adult Patients in Intensive Care Unit (FUNDICU) 2024 consensus, and Bulpa criteria (Bulpa et al., 2007; Donnelly et al., 2020; Bassetti et al., 2024).

A total of 289 IPA patients were identified from multiple departments including Respiratory and Critical Care Medicine, Hematology, Rheumatology and Immunology, and ICU. Of them, 278 eligible cases were ultimately included after excluding 3 cases with incomplete data of Serum GM and CBC-derived inflammatory biomarkers, and 8 cases with severe immunosuppression (lymphocyte count of 0 or neutrophil count less than 0.5×10^9^/L). The flowchart was presented in **Supplementary Figure 1**. In-hospital death occurred in 79 patients. This study has been approved by the Ethics Committee of Nanjing Drum Tower Hospital (2023-503-02) and adheres to the principles of the Declaration of Helsinki.

### 2.2 Data collection and preprocessing

All data acquisition was authorized by the relevant departments of the hospital. Clinical information, including demographic information, diagnosis information, history of present illness, past medical history, imaging examination, and laboratory test results were extracted from the electronic medical record system. All data were deep-cleaned and manually checked before forming a standardized specialized disease database before statistical analysis.

Patients in critical condition were defined as those with a continuously adverse progression of their condition and who presented with the following conditions: admission to ICU, mechanical ventilation, severe pneumonia, sepsis/septic shock, or acute respiratory distress syndrome. Information on prognosis was gathered based on the professional assessments of researchers. Patients were grouped into survival or non-survival groups depending on the occurrence of an in-hospital death. Additionally, we acquired and verified the survival status of patients within 30 days and 90 days via telephone follow-up and medical records.

### 2.3 Serum GM and CBC-derived inflammatory biomarkers

All laboratory tests were conducted in the Department of Laboratory Medicine in Nanjing Drum Tower Hospital. Serum GM and CBC samples were collected at the time of IPA diagnosis or prior to initiation of antifungal therapy.

Serum GM was initial treated as a continuous variable to capture the full range of fungal burden. The cut-off value of 0.7 was data-driven, determined from the ROC curve using the Youden index (see Results 3.2). CBC-derived inflammatory biomarkers, including the NLR, platelet-to-lymphocyte ratio (PLR), monocyte-to-lymphocyte ratio (MLR), neutrophil-monocyte-to-lymphocyte ratio (NMLR), systemic inflammation response index (SIRI), and systemic immune-inflammation index (SII), reflect the immune-inflammatory status and serve as reliable predictive indicators for various inflammatory and infectious diseases (Raffetti et al., 2015; Asperges et al., 2023; Li et al., 2023a; Cannata et al., 2025). The formulas used to calculate these indices: NLR = neutrophils counts (10^9^/L)/lymphocytes counts (10^9^/L); PLR = platelets counts (10^9^/L)/lymphocytes counts (10^9^/L); MLR = monocytes counts (10^9^/L)/lymphocytes counts (10^9^/L); NMLR = (monocytes counts (10^9^/L) + neutrophils counts (10^9^/L))/lymphocytes counts (10^9^/L); SIRI = neutrophils counts (10^9^/L) × monocytes counts (10^9^/L)/lymphocytes counts (10^9^/L); SII = platelets counts (10^9^/L) × neutrophils counts (10^9^/L)/lymphocytes counts (10^9^/L).

### 2.4 Model training, evaluation, and application

The study was designed to develop a predictive model for in-hospital mortality among non-neutropenic IPA patients. We initially compared six CBC-derived inflammatory biomarkers, and as NLR demonstrated the best overall predictive performance in terms of area under the curve (AUC), sensitivity, and specificity, it was selected for inclusion in the final prognostic model (see Results 3.3). Based on the predictive significance of serum GM and NLR, we additionally incorporated age and critical condition as the central predictive factors. A multivariate logistic regression analysis was employed to formulate the predictive model, and internal validation was carried out via the Bootstrap resampling with 1000 replications. Model performance was evaluated on discrimination, calibration and clinical applicability, and a clinical nomogram was constructed for decision-making.

### 2.5 Statistical analysis

All statistical analyses were performed using R (version 4.2.1). Data distribution was assessed using the Shapiro–Wilk test. Continuous variables were presented as mean ± SD for normal distributions or median (Q1, Q3) for non-normal distributions. Categorical variables are reported in terms of frequency (n) and proportion (%). For normally distributed continuous variables, Student’s t-test was used if the variance between groups was equal, whereas Welch’s t-test was applied if the variance was unequal. Non-normally distributed continuous variables were compared using the Mann–Whitney U test. Categorical variables were compared using the chi-squared test or Fisher’s exact test as appropriate. Group comparisons of categorical variables were performed using Chi-square test with *post-hoc* Bonferroni correction for multiple comparisons where appropriate. The predictive performance of serum GM and CBC-derived inflammatory biomarkers was evaluated by receiver operating characteristic (ROC) analysis respectively, with the optimal cut-off determined by Youden’s index. Restricted cubic splines (RCS) were applied to explore their associations with in-hospital mortality. Collinearity analysis was performed among inflammatory biomarkers (Spearman correlation coefficients available in **Supplementary Figure 2**), and the optimal was chosen based on the discriminatory capacity for the prediction of in-hospital mortality. The 30-day and 90-day survival curves were plotted using the Kaplan-Meier method, and the Log-rank test was applied to compare the differences among groups. P < 0.05 indicated statistically significant.

## 3 Results

### 3.1 Demographic and clinical characteristics of IPA patients

A total of 278 eligible patients with IPA (5 proven, 258 probable, and 15 possible) were included in the study, with 199 in the survival group and 79 in the non-survival group. The in-hospital mortality among IPA patients was 28.4% (79/278), with patients in critical condition experiencing a significantly higher rate of 35.2% (77/219) compared to merely 3.4% (2/59) in non-critical condition (P < 0.001, **Supplementary Figure 3**).

Demographic information, host factors, clinical manifestations, and laboratory tests between the survival group and death group were presented in **Table 1**. Compared with the survival group, the non-survival group had a higher median age (72.00 vs. 68.00), a greater proportion of patients in critical condition (97.5% vs. 71.4%), a higher prevalence of respiratory failure (95.0% vs. 54.5%) and symptoms of dyspnea (89.9% vs. 73.9%, P = 0.001), and a significantly higher proportion of individuals receiving mechanical ventilation (81.0% vs. 20.1%, P < 0.001). The most common host factors were the application of immunosuppressants (including long-term or high-dose administration of glucocorticoids), diabetes, and chronic lung diseases. All patients underwent anti-*Aspergillus* treatment, among which 65 cases (23.4%) received combination therapy with two or more anti-*Aspergillus* drugs. Notably, seven patients received nebulized liposomal amphotericin B as a component of their regimen: five in combination with triazoles plus echinocandins, and two with triazoles plus intravenous amphotericin B. Further comparison of co-infection and anti-*Aspergillus* treatment between the survival and non-survival groups (**Supplementary Table 1**) showed significant differences in co-infection rates (χ² = 46.684, P < 0.001). The proportion of multiple co-infections involving bacteria, viruses or other fungi (excluding *Aspergillus*) was higher in the non-survival group (27.8% vs. 6.0%). The most common co-infection in the survival and non-survival group was bacterial infection (44.7% and 60.8%). Combination therapy was used in 21.1% and 29.1% patients, respectively. The difference in treatment approach between the survival and non-survival was not statistically significant (χ² = 2.025, P = 0.155).

**Table 1:** The clinical characteristics between the survival and non-survival group^*^ of IPA.

n	Overall	Survival	Non-survival	P-value	SMD
	278	199	79		
Gender = Male (%)	202 (72.7)	148 (74.4)	54 (68.4)	0.310	0.133
Age (median [IQR])	69.00 [59.25, 74.00]	68.00 [58.00, 73.00]	72.00 [65.00, 79.00]	0.001	0.334
Smoking (%)	109 (39.2)	82 (41.2)	27 (34.2)	0.279	0.145
Diagnosis (%)				0.370	0.204
Proven	5 (1.8)	4 (2.0)	1 (1.3)		
Probable	258 (92.8)	182 (91.5)	76 (96.2)		
Possible	15 (5.4)	13 (6.5)	2 (2.5)		
Host factors					
Chronic lung diseases					
COPD (%)	66 (23.7)	50 (25.1)	16 (20.3)	0.389	0.117
ILD (%)	85 (30.6)	56 (28.1)	29 (36.7)	0.162	0.184
Lung cancer (%)	17 (6.1)	15 (7.5)	2 (2.5)	0.116	0.230
Bronchiectasis (%)	32 (11.5)	28 (14.1)	4 (5.1)	0.034	0.310
Respiratory Virus Infection (%)	64 (23.0)	41 (20.6)	23 (29.1)	0.128	0.198
Extrapulmonary diseases					
Hematological malignancies (%)	1 (0.4)	0 (0.0)	1 (1.3)	0.112	0.160
Kidney diseases (%)	14 (5.0)	8 (4.0)	6 (7.6)	0.219	0.153
Cirrhosis (%)	8 (2.9)	3 (1.5)	5 (6.3)	0.030	0.250
Diabetes (%)	91 (32.7)	61 (30.7)	30 (38.0)	0.241	0.155
Autoimmune diseases (%)	54 (19.4)	36 (18.1)	18 (22.8)	0.372	0.117
Solid tumor^a^ (%)	27 (9.7)	21 (10.6)	6 (7.6)	0.453	0.103
Immunosuppressants^b^ (%)	134 (48.2)	92 (46.2)	42 (53.2)	0.297	0.139
Clinical symptoms					
Fever (%)	199 (71.6)	143 (71.9)	56 (70.9)	0.871	0.022
Cough (%)	258 (92.8)	188 (94.5)	70 (88.6)	0.088	0.212
Expectoration (%)	245 (88.1)	182 (91.5)	63 (79.7)	0.006	0.338
Hemoptysis (%)	45 (16.2)	38 (19.1)	7 (8.9)	0.037	0.298
Chest tightness (%)	165 (59.4)	110 (55.3)	55 (69.6)	0.028	0.300
Dyspnea (%)	218 (78.4)	147 (73.9)	71 (89.9)	0.003	0.425
Chest pain (%)	16 (5.8)	11 (5.5)	5 (6.3)	0.796	0.034
Severity					
Critical condition^c^ (%)	219 (78.8)	142 (71.4)	77 (97.5)	<0.001	0.772
Respiratory failure (%)				<0.001	1.067
Type I	145 (52.3)	89 (44.9)	56 (70.9)		
Type II	38 (13.7)	19 (9.6)	19 (24.1)		
Mechanical ventilation (%)	104 (37.4)	40 (20.1)	64 (81.0)	<0.001	1.536
Co-infection (%)	216 (77.7)	140 (70.4)	76 (96.2)	<0.001	0.738
Combination anti-Aspergillus therapy (%)	65 (23.4)	42 (21.1)	23 (29.1)	0.155	0.185
Aspergillus type (%)				0.139	0.421
Fumigatus	113 (61.7)	74 (57.4)	39 (72.2)		
Flavus	50 (27.3)	37 (28.7)	13 (24.1)		
Niger	14 (7.7)	13 (10.1)	1 (1.9)		
Other	6 (3.3)	5 (3.9)	1 (1.9)		
Mycological tests					
Sputum Aspergillus culture (%)	111/271 (41.0)	71/195 (36.4)	40/76 (52.6)	0.015	0.331
BALF Aspergillus culture (%)	56/196 (28.6)	36/141 (25.5)	20/55 (36.4)	0.132	0.236
Serum GM (median [IQR])	0.24 [0.10, 1.00]	0.19 [0.10, 0.48]	1.00 [0.16, 3.94]	<0.001	0.807
Serum GM ≥ 1.0 (%)	72/278 (25.9)	31/199 (15.6)	41/79 (51.9)	<0.001	0.832
BALF GM (median [IQR])	2.16 [0.69, 4.20]	1.53 [0.45, 3.92]	3.95 [1.64, 5.03]	<0.001	0.695
BALF GM ≥ 1.0 (%)	133/193 (68.9)	85/137 (62.0)	48/56 (85.7)	0.001	0.560
Aspergillus-specific IgG (median [IQR])	140.47 [84.04, 348.77]	127.71 [80.19, 336.91]	305.06 [112.44, 377.30]	0.294	0.231
CBC-derived inflammatory biomarkers					
NLR (median [IQR])	7.84 [3.94, 17.11]	6.36 [3.45, 13.67]	15.17 [7.33, 30.12]	<0.001	0.544
PLR (median [IQR])	225.22 [137.64, 377.56]	216.67 [136.48, 369.58]	260.00 [139.38, 387.50]	0.548	0.122
MLR (median [IQR])	0.50 [0.31, 0.70]	0.50 [0.30, 0.67]	0.50 [0.36, 1.00]	0.027	0.308
NMLR (median [IQR])	8.37 [4.53, 17.96]	6.88 [3.94, 14.17]	15.60 [7.69, 30.90]	<0.001	0.546
SIRI (median [IQR])	2.96 [1.48, 6.49]	2.55 [1.29, 4.77]	5.73 [2.17, 11.73]	<0.001	0.354
SII (median [IQR])	1572.94 [721.55, 3328.47]	1385.13 [646.71, 2664.06]	2399.33 [1002.10, 4338.21]	0.002	0.309
Inflammatory markers					
C-reactive protein, mg/L (median [IQR])	98.05 [41.85, 170.40]	78.00 [32.20, 138.20]	150.10 [89.70, 241.25]	<0.001	0.680
Procalcitonin, ng/mL (median [IQR])	0.25 [0.09, 1.56]	0.15 [0.07, 0.63]	1.62 [0.31, 6.63]	<0.001	0.408
Interleukin-6, pg/mL (median [IQR])	81.52 [21.94, 289.53]	49.92 [14.99, 123.17]	294.28 [97.69, 1198.28]	<0.001	0.339
Heparin binding protein, ng/mL (median [IQR])	80.70 [32.26, 153.08]	62.77 [31.64, 137.44]	101.51 [46.47, 174.49]	0.152	0.130

^*^Patients were grouped into survival or non-survival groups depending on the occurrence of an in-hospital death.

a Solid tumor treated, diagnosed or in complete remission for less than 5 years.

b Immunosuppressive agents or prednisone equivalent ≥ 0.5 mg/kg/d or ≥ 3 months.

c Patients with a continuously adverse progression of their condition and who presented with the following conditions: admission to ICU, mechanical ventilation, severe pneumonia, sepsis/septic shock, or acute respiratory distress syndrome.

COPD, chronic obstructive pulmonary disease; ILD, interstitial lung disease; BALF, bronchoalveolar lavage fluid; GM, galactomannan; NLR, neutrophil-to-lymphocyte ratio; PLR, platelet-to-lymphocyte ratio; MLR, monocyte-to-lymphocyte ratio; NMLR, neutrophil-monocyte-to-lymphocyte ratio; SIRI, systemic inflammation response index; SII, systemic immune-inflammation index.

The positive rate of bronchoalveolar lavage fluid (BALF) GM (cut-off = 1.0) test was as high as 68.9% (133/193), markedly exceeding the 25.9% (72/278) positive rate observed in serum GM tests (cut-off = 1.0). The GM values were significantly higher in the death group compared to the survival group (P < 0.001). **Supplementary Table 2** showed the cross-tab of BALF GM and serum GM with in-hospital mortality. Patients positive for both markers had the highest mortality (25/37), while those negative for both had the lowest (6/51). Because BALF GM had approximately 30% missing data, it was excluded from further prognostic analysis. Inflammatory markers, including CRP, procalcitonin, Interleukin-6, as well as CBC-derived inflammatory biomarkers were also observed as higher levels in the non-survival group.

Furthermore, in univariate logistic regression, age (per 10 years) was associated with an increased risk of in-hospital mortality (OR = 1.347, 95% CI: 1.076-1.713, P = 0.012). Patients in critical condition had a markedly higher risk of in-hospital death (OR = 15.454, 95% CI: 4.643-95.905, P < 0.001) (**Supplementary Table 3**).

### 3.2 Prognostic performance of serum GM in IPA patients

**Figure 1:**
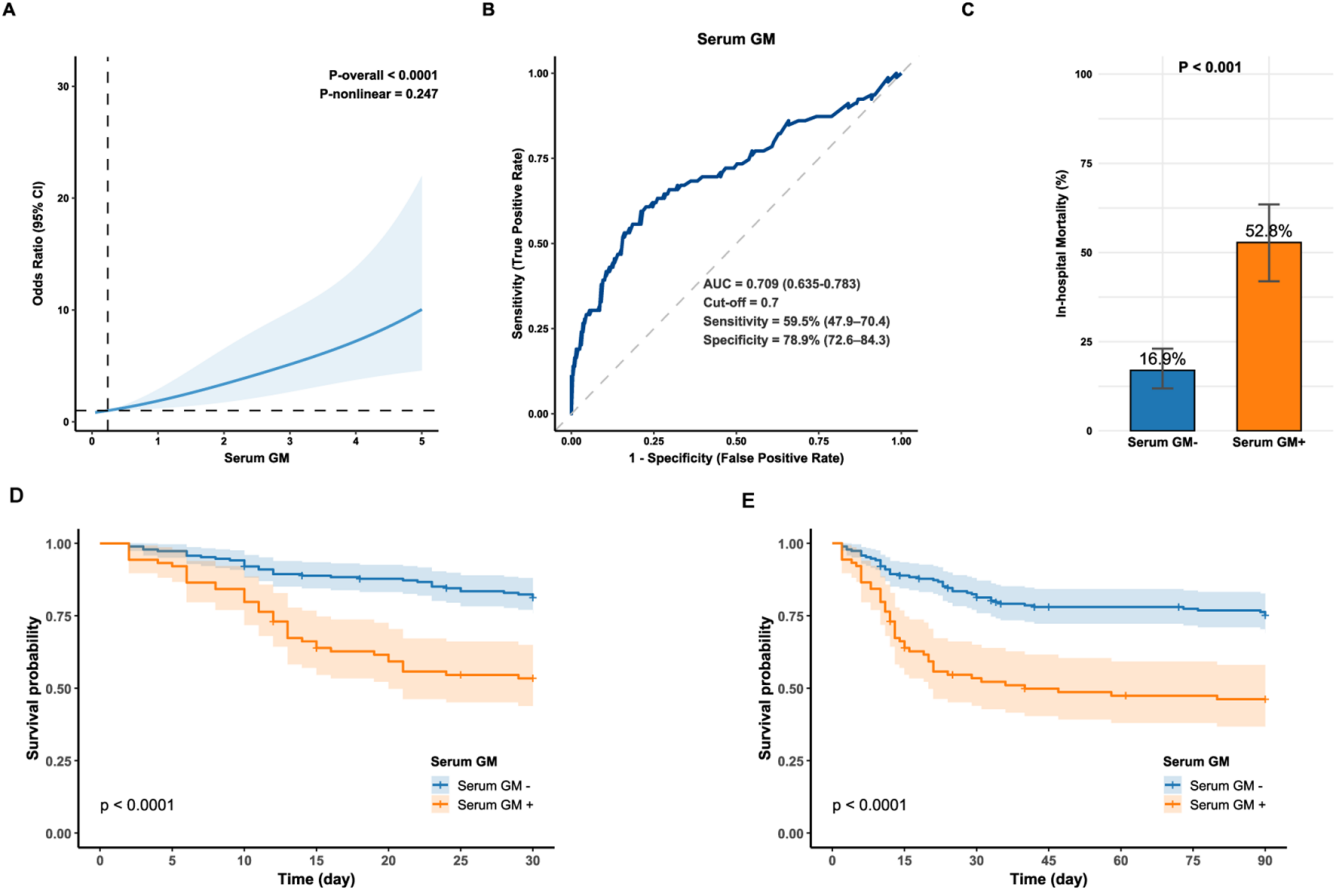
**(A)** Restricted cubic spline for the association between serum GM levels and the odds of in-hospital mortality in IPA; **(B)** Receiver operator characteristic curve of serum GM for predicting in-hospital mortality in IPA (optimal cut-off value = 0.7); **(C)** In-hospital mortality between the Serum GM- and Serum GM+ group. Statistical significance was assessed with Chi-square test. **(D, E)** Kaplan-Meier survival curve compares 30-day and 90-day survival between the Serum GM- and Serum GM+ group (Log-rank, P < 0.0001). Serum GM-: serum GM < 0.7; Serum GM+: serum GM ≥0.7. AUC, area under the curve; GM, galactomannan; IPA, invasive pulmonary aspergillosis.

As presented in **Supplementary Figure 4**, the non-survival group exhibited a significantly higher median serum GM level compared to the survival group (1.00 [0.16, 3.94] vs. 0.19 [0.10, 0.48]; P < 0.001). RCS analysis indicated a linear positive association between serum GM and in-hospital mortality risk (P-overall < 0.001; P-nonlinear = 0.247; **Figure 1A**). ROC curve analysis identified serum GM as a moderate predictor of mortality, achieving an AUC of 0.709 (95% CI: 0.635–0.783; **Figure 1B**). Using the Youden index-derived optimal cut-off value of 0.7, serum GM provided a specificity of 78.9% (95% CI: 72.6–84.3%), but a low sensitivity of 59.5% (95% CI: 47.9–70.4%). Bootstrap resampling (1,000 iterations) confirmed the optimal cut-off of 0.7 (95% CI: 0.3–1.4). At the threshold of 0.7, there were 47 true positives (TP), 42 false positives (FP), 157 true negatives (TN), and 32 false negatives (FN), yielding a positive predictive value (PPV) of 52.8% (95% CI: 41.9–63.5%) and a negative predictive value (NPV) of 83.1% (95% CI: 76.9–88.1%) (**Supplementary Table 4**). With alternative thresholds, sensitivity and specificity varied: at 0.5, sensitivity was 62.0% (95% CI: 50.4–72.7%) and specificity was 74.9% (95% CI: 68.3–80.7%); at 1.0, sensitivity decreased to 51.9% (95% CI: 40.4–63.3%) and specificity increased to 84.4% (95% CI: 78.6–89.2%) (**Supplementary Table 5**).

Moreover, patients with serum GM ≥ 0.7 had a three-fold higher mortality risk than those below this threshold (52.8% vs. 16.9%; P < 0.001; **Figure 1C**) and lower 30- and 90-day survival (Log-rank P < 0.0001; **Figures 1D, E**), highlighting its clinical relevance. Consistently, cox regression analysis showed that for 30-day mortality, serum GM had a Hazard Ratio (HR) of 1.375 (95% CI: 1.256–1.505, P < 0.001) as a continuous variable, and 3.093 (95% CI: 1.968–4.863, P < 0.001) as a categorical variable (serum GM ≥ 0.7). For 90-day mortality, the HRs were 1.350 (95% CI: 1.238–1.472, P < 0.001) and 2.829 (95% CI: 1.881–4.254, P < 0.001), respectively (**Supplementary Table 6**).

### 3.3 Prognostic performance of CBC-derived inflammatory biomarkers in IPA patients

Mann-Whitney U analysis revealed significantly elevated levels of CBC-derived inflammatory biomarkers in the death group compared to the survival, with the exception of PLR (summarized in **Table 1**). Biomarkers demonstrating statistical significance included NLR (15.17 [7.33, 30.12] vs. 6.36 [3.45, 13.67]; P < 0.001), MLR (0.50 [0.36, 1.00] vs. 0.50 [0.30, 0.67]; P = 0.027), NMLR (15.60 [7.69, 30.90] vs. 6.88 [3.94, 14.17]; P < 0.001), SIRI (5.73 [2.17, 11.73] vs. 2.55 [1.29, 4.77]; P < 0.001) and SII (2399.33 [1002.10, 4338.21] vs. 1385.13 [646.71, 2664.06]; P = 0.002).

RCS analyses (**Supplementary Figure 5**) delineated that NLR (*P*-overall < 0.0001, *P*-nonlinear = 0.002), NMLR (P-overall < 0.0001, P-nonlinear = 0.002), and SIRI (P-overall < 0.001, P-nonlinear = 0.001) exhibited nonlinear positive associations with the odds of in-hospital mortality with a gradually saturating trend. MLR (P-overall = 0.032, P-nonlinear = 0.281) and SII (P-overall = 0.013, P-nonlinear = 0.063) revealed positive near-linear associations with the odds of in-hospital mortality. PLR remained non-significant associated with the odds of in-hospital mortality (P-overall = 0.740, P-nonlinear = 0.859), further validating its limited prognostic utility.

As illustrated in **Supplementary Figure 6**, ROC curve analyses evaluated the prognostic performance of six inflammatory biomarkers. Detailed diagnostic performance, including AUC, optimal cut-off values, sensitivity, specificity, positive/negative predictive values, accuracy, and Youden index, were comprehensively summarized in **Supplementary Table 7**. Among all indicators, only NLR and NMLR demonstrated moderate discriminative abilities, with AUC values above 0.7. In the collinearity analysis within the methodology section, we detected perfect collinearity between the NLR and the NMLR (ρ = 1.00, P < 0.001). Given the broader application of NLR in clinical practice and its relatively straightforward calculation, this research selected NLR as a prognostic indicator for subsequent analyses, which was significantly elevated in the non-survival (**Supplementary Figure 7**). At the optimal cut-off value of 8.6, the sensitivity of NLR in predicting in-hospital mortality was 70.9% (95% CI: 59.6 - 80.6%), and the specificity was 63.3% (95% CI: 56.2 - 70.0%).

A positive correlation was observed between NLR and CRP (ρ = 0.46, P < 0.001) (**Supplementary Figure 8**). ROC analysis showed that CRP as a continuous predictor had an AUC of 0.697 (95% CI: 0.628–0.765). The optimal cutoff of 79.8 mg/L provided 81.0% sensitivity (95% CI: 70.6–89.0%) and 50.3% specificity (95% CI: 43.1–57.4%). (**Supplementary Figure 9**). Similarly, elevated CRP and NLR levels were associated with higher 30-day and 90-day mortality (**Supplementary Figure 10**).

After applying Bonferroni correction, the chi-square test analysis demonstrated that CRP exhibited the highest sensitivity for predicting in-hospital mortality, significantly surpassing serum GM (81.0% vs. 59.5%, P-adjusted = 0.015). However, no statistically significant difference was observed between CRP and NLR (81.0% vs. 70.9%, P-adjusted = 0.577). Additionally, serum GM showed the highest specificity, which was significantly greater than that of NLR (78.9% vs. 63.3%, P-adjusted = 0.003) and CRP (78.9% vs. 50.3%, P < 0.001). The specificity of NLR was also significantly greater than that of CRP (63.3% vs. 50.3%, P-adjusted = 0.034) (**Supplementary Figure 11**). Given that NLR and CRP were correlated, NLR was chosen for further prognostic analysis due to its superior specificity.

### 3.4 Synergistic prognostic value of combined serum GM and NLR for in-hospital mortality in IPA patients

Serum GM exhibited relatively higher specificity for prognostic prediction, whereas the NLR demonstrated superior sensitivity. Owing to their complementary predictive performance, combining serum GM (cut-off = 0.7) with NLR (cut-off = 8.6) was hypothesized to enhance prognostic prediction. Therefore, samples were stratified into three groups based on this complementary principle: (a) dual-positive group: serum GM + & NLR + (n=58); (b) single positive group: serum GM +/NLR + (n=102); (c) dual-negative group: GM- & NLR- (n=118). Significant mortality gradients across these groups were observed in both overall cohorts and subgroup analyses.

**Figure 2:**
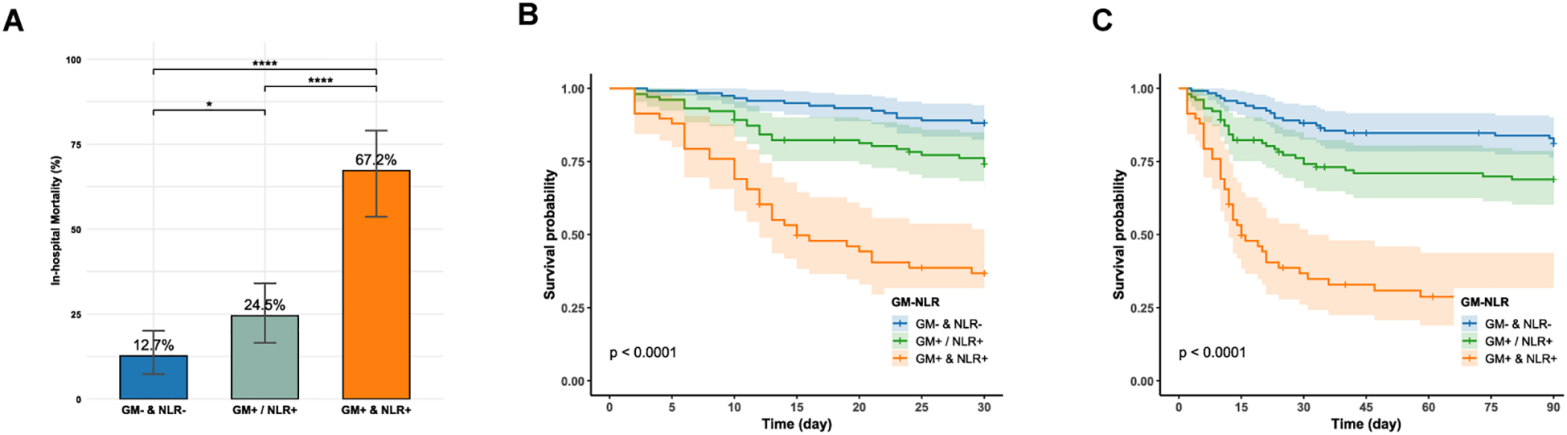
**(A)** In-hospital mortality between the dual-negative, single-positive, and dual-positive groups; GM: galactomannan; NLR: neutrophil-to-lymphocyte ratio. **(B, C)** Kaplan-Meier survival curve compares 30-day and 90-day survival between the dual-negative, single-positive, and dual-positive groups (Log-rank, P < 0.0001). GM-: serum GM < 0.7; GM+: serum GM ≥0.7; NLR-: NLR < 8.6; NLR+: NLR ≥ 8.6; ^****^P < 0.0001; ^*^P < 0.05.

As illustrated in **Figures 2A**, the dual-positive group demonstrated the highest in-hospital mortality of 67.2% (39/58), showing statistically significant differences compared to both single-positive (25/102) (67.2% vs. 24.5%, P < 0.0001) and dual-negative groups (15/118) (67.2% vs. 12.7%, P < 0.0001). Notably, the single-positive group also exhibited significantly higher mortality than the dual-negative group (24.5% vs. 12.7%, P = 0.037), suggesting synergistic prognostic prediction through biomarker combination. After adjusting for age, critical condition, treatment, and co-infection, the dual-positive group remained independently associated with increased in-hospital mortality compared with the dual-negative group (OR = 12.289, 95% CI: 5.308-30.418, P < 0.001) (**Supplementary Table 8**). Kaplan-Meier analysis also found that the dual-positive group suffered significantly lower 30-day and 90-day survival (Log-rank test, P < 0.0001, **Figures 2B, C**). Furthermore, the dual-positive group had significantly higher in-hospital mortality across all subgroups, including critical and non-critical patients, ICU and non-ICU patients, and those with or without COPD (**Supplementary Table 9**).

As detailed in **Table 2**, the combination strategies showed distinct prediction performance. The combination of either positive for serum GM or NLR (satisfying any one of the following conditions: serum GM+ & NLR+, serum GM+ & NLR-, and serum GM- & NLR+) exhibited a sensitivity of 81.0%, which was higher than those of serum GM (81.0% vs. 59.5%, P = 0.005). However, this was accompanied by a reduction in specificity, with values of 51.8%. Conversely, the dual-positive combination of serum GM and NLR achieved a specificity of 90.5%, which was markedly higher than those of serum GM or NLR alone (90.5% vs. 78.9% for serum GM, P = 0.002; 90.5% vs. 63.3% for NLR, P < 0.001). However, this improvement in specificity came at the expense of reduced sensitivity of 49.4%. Notably, the dual-positive strategy also demonstrated the highest overall diagnostic accuracy (78.8%).

**Table 2:** Prognostic performance of serum GM, NLR, and combined indicators.

Indicator	Sensitivity (%)	Specificity (%)	Positive predictive value	Negative predictive value	Accuracy
Serum GM (cut-off=0.7)	59.5% (47.9–70.4)	78.9% (72.6–84.3)	52.8% (41.9–63.5)	83.1% (76.9–88.1)	73.4% (67.8–78.5)
NLR (cut-off=8.6)	70.9% (59.6–80.6)	63.3% (56.2–70.0)	43.4% (34.7–52.4)	84.6% (77.7–90.0)	65.5% (59.6–71.0)
Serum GM or NLR	81.0% (70.6–89.0)	51.8% (44.6–58.9)	40.0% (32.3–48.0)	87.3% (79.9–92.7)	60.1% (54.1–65.9)
Serum GM and NLR	49.4% (37.9–60.9)	90.5% (85.5–94.2)	67.2% (53.7–79.0)	81.8% (76.1–86.7)	78.8% (73.5–83.4)

GM, galactomannan; NLR, neutrophil-to-lymphocyte ratio.

### 3.5 Construction, validation and visualization of the predictive model

**Figure 3:**
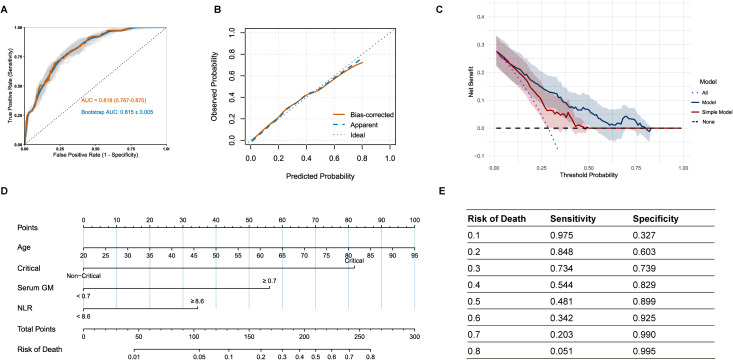
**(A)** Receiver operating characteristic curve of the model on the original data and bootstraps validation; **(B)** Calibration curve with bootstrap resampling of the model; **(C)** Decision curve analysis with bootstrap resampling of the model and simple model containing only age and critical condition; **(D)** Nomogram for the risk of in-hospital mortality in IPA patients; **(E)** The sensitivity and specificity corresponding to various mortality risk values. IPA: invasive pulmonary aspergillosis; patients in critical condition were defined as those with a continuously adverse progression of their condition and who presented with the following conditions: admission to ICU, mechanical ventilation, severe pneumonia, sepsis/septic shock, or acute respiratory distress syndrome.

Based on the established predictive value of the serum GM and NLR, we developed a comprehensive risk prediction model for in-hospital mortality in IPA patients by integrating additional factors of age and critical condition. The final multivariable logistic regression model encompasses four independent predictive factors (**Supplementary Table 10**). Following internal validation through bootstrap resampling with 1000 iterations, the model demonstrated robust discriminative ability with a mean AUC of 0.815 (SD = 0.005). This result closely aligned with the original model AUC of 0.818 (95% CI: 0.767-0.870), indicating excellent stability (**Figure 3A**). The calibration curve also showed excellent agreement between predicted and observed mortality risks, approximating the ideal line (**Figure 3B**). Furthermore, decision curve analysis with bootstrap resampling confirmed the clinical applicability of the comprehensive model, as it yielded a higher net benefit compared to the simple model that included only age and critical condition (**Figure 3C**; **Supplementary Table 11**), highlighting the added value of integrating serum GM and NLR biomarkers into clinical decision-making.

A clinical nomogram was developed based on the multivariate logistic regression analysis (**Figure 3D**), in which the “Total Points” represented the cumulative scores of individual variables, and the “Risk of Death” indicated the corresponding predicted mortality risk. **Figure 3E** demonstrated the sensitivity and specificity of the nomogram across mortality risk thresholds, assisting in clinical decision-making.

## 4 Discussion

Despite receiving standardized antifungal therapy, IPA patients continue to exhibit high mortality due to disease deterioration (Hoang et al., 2022), indicating that both the immune-inflammatory status of the host and the fungal load jointly influence the prognosis (Feys et al., 2025). The study retrospectively analyzed 278 non-neutropenic IPA patients admitted to various departments, of whom 79 (28.4%) died during hospitalization. Our study provided novel evidence that the synergistic integration of mycological (serum GM) and inflammatory biomarkers (NLR) significantly improves prognostic accuracy in non-neutropenic IPA. The developed clinical nomogram offers a practical tool to support clinical decision-making.

It has been demonstrated that the dynamic changes in serum GM were closely associated with the treatment outcomes of hematology patients with IPA (Mercier et al., 2020). However, its value in non-neutropenic IPA patients remains unclear. In this study, serum GM exhibited a high specificity (78.9%) but low sensitivity (59.5%) for predicting in-hospital mortality, suggesting that relying on it alone may miss nearly 40% of high-risk cases. In recent years, CBC-derived inflammatory biomarkers have emerged as novel predictors for the prognosis of infectious diseases (Russell et al., 2019). An exploratory study suggested that NLR may predict mortality or treatment response in heart transplant recipients (Urbanowicz et al., 2021). However, it focused on a narrow population and did not incorporate mycologist testing. We examined the association between NLR and in-hospital mortality in a broader cohort of non-neutropenic IPA patients, incorporating serum GM to integrate both pathogen- and host-centered perspectives, offering a novel prognostic framework.

The study reported that the NLR exhibited relatively higher sensitivity of 70.9%, which was not statistically different from that of CRP (70.9% vs. 81.0%, P-adjusted = 0.577), but was significantly higher than that of serum GM (70.9% vs. 59.5%, P-adjusted = 0.015). When serum GM and NLR were used in combination, the accuracy of prognosis prediction was significantly improved. The double-positive status (serum GM+ and NLR+) showed a significantly higher specificity of 90.5%, suggesting that antifungal treatment escalation and immunomodulatory interventions should be prioritized for these patients. Meanwhile, the parallel strategy (serum GM+ or NLR+) demonstrated a sensitivity of 81.0%, making it suitable for screening high-risk patients. The final comprehensive model, integrating age, critical condition, serum GM, and NLR, demonstrated robust discrimination with a mean AUC of 0.815 (SD = 0.005) after bootstrap resampling with 1000 iterations. The result closely matched the original model AUC of 0.818 (95% CI: 0.767-0.870). Decision curve analysis further confirmed that the comprehensive model provided a higher net benefit than the simple model based solely on age and critical condition, highlighting the clinical utility of including serum GM and NLR as biomarkers in decision-making processes.

All patients are exposed to spores; however, in a normal host response, conidia are eliminated before pathogenesis, preventing excessive inflammation (Ledoux and Herbrecht, 2023). Neutrophils play a crucial role against fungi through multiple mechanisms, including phagocytosis, oxidative burst and the formation of neutrophil extracellular traps (Alflen et al., 2020; Feys et al., 2022; Salazar et al., 2022; Wang et al., 2024b). These first-line effector cells further trigger adaptive immune responses and induce Th1 cell responses, accompanied by the augmented secretion of interferon, Interleukin-2 and Interleukin-12 as well as the activation and proliferation of lymphocyte (Beisswenger et al., 2012). The balance between neutrophils and lymphocyte is therefore essential for pathogen clearance while avoiding excessive tissue injury, and the NLR has emerged as a simple marker of such immune imbalance (Kim et al., 2018; Wang et al., 2024a). Building on our previous finding that elevated Pentraxin-3 predicts poor outcomes in non-neutropenic IPA, this study shows that NLR also reflects dysregulated host responses, providing complementary prognostic value by integrating immune and pathogen factors (Sun et al., 2025).

This study still has several limitations. Firstly, the present study is limited by its single-time-point detection method, which prevents the observation of the dynamic changes in serum GM and NLR and their associations with treatment responses. Moreover, the absence of a multicenter external validation cohort underscores the necessity for larger, prospective studies to further validate the prognostic value of the model based on serum GM and NLR.

In the future, we propose to further illuminate the immune-fungal interaction mechanism of IPA via multi-omics analysis, and establish a multimodal prognostic model by integrating host factors, radiomics, and biomarkers. Eventually, we will undertake clinical intervention trials to accomplish the transformation closed loop from mechanism exploration to precise intervention, and systematically advance the individualized process of IPA treatment.

## Data Availability

The raw data supporting the conclusions of this article will be made available by the authors, without undue reservation.

## Glossary

AUC: area under the curve
BALF: bronchoalveolar lavage fluid
CBC: complete blood cell count
CI: confidence intervals
CRP: C-reactive protein
COPD: chronic obstructive pulmonary disease
EORTC/MSGERC: European Organization for Research and Treatment of Cancer and the Mycoses Study Group Education and Research Consortium
FN: false negatives
FP: false positives
GM: galactomannan
HR: Hazard Ratio
ICU: intensive care unit
IPA: Invasive pulmonary aspergillosis
MLR: monocyte-to-lymphocyte ratio
NPV: negative predictive value
NMLR: neutrophil-monocyte-to-lymphocyte ratio
NLR: neutrophil-to-lymphocyte ratio
PLR: platelet-to-lymphocyte ratio
PPV: positive predictive value
SIRI: systemic inflammation response index
SII: systemic immune-inflammation index
TN: true negatives
TP: true positives
OR: odds ratios
RCS: restricted cubic splines
ROC: receiver operating characteristic.
