# Supplementary material for: A serum galactomannan and neutrophil-to-lymphocyte ratio-based nomogram for predicting in-hospital mortality in non-neutropenic invasive pulmonary aspergillosis
Source: Front Cell Infect Microbiol. 2025 Oct 22;15:1675277. doi: 10.3389/fcimb.2025.1675277 (PMC12585980; doi:10.3389/fcimb.2025.1675277)
Supplement: Supplementary file 1 [file Table1.docx]

A Serum Galactomannan and Neutrophil-to-Lymphocyte Ratio-Based Nomogram for Predicting In-Hospital Mortality in Non-Neutropenic Invasive Pulmonary Aspergillosis

Xinyu Wang, Wenjuan Li, Yunyu Ma, Yi Li, ShenYan Ding, Jiayi Shen, Tingting Zhao, Yajie Lu, Chao Sun, Xin Su*

[Supplementary Figure 1 3](#_Toc209700524)

[Supplementary Figure 2 4](#_Toc209700525)

[Supplementary Figure 3 5](#_Toc209700526)

[Supplementary Figure 4 6](#_Toc209700527)

[Supplementary Figure 5 7](#_Toc209700528)

[Supplementary Figure 6 8](#_Toc209700529)

[Supplementary Figure 7 9](#_Toc209700530)

[Supplementary Figure 8 10](#_Toc209700531)

[Supplementary Figure 9 11](#_Toc209700532)

[Supplementary Figure 10 12](#_Toc209700533)

[Supplementary Figure 11 13](#_Toc209700534)

[Supplementary Table 1 14](#_Toc209700535)

[Supplementary Table 2 15](#_Toc209700536)

[Supplementary Table 3 16](#_Toc209700537)

[Supplementary Table 4 17](#_Toc209700538)

[Supplementary Table 5 18](#_Toc209700539)

[Supplementary Table 6 19](#_Toc209700540)

[Supplementary Table 7 20](#_Toc209700541)

[Supplementary Table 8 21](#_Toc209700542)

[Supplementary Table 9 22](#_Toc209700543)

[Supplementary Table 10 23](#_Toc209700544)

[Supplementary Table 11 24](#_Toc209700545)

# Supplementary Figure 1


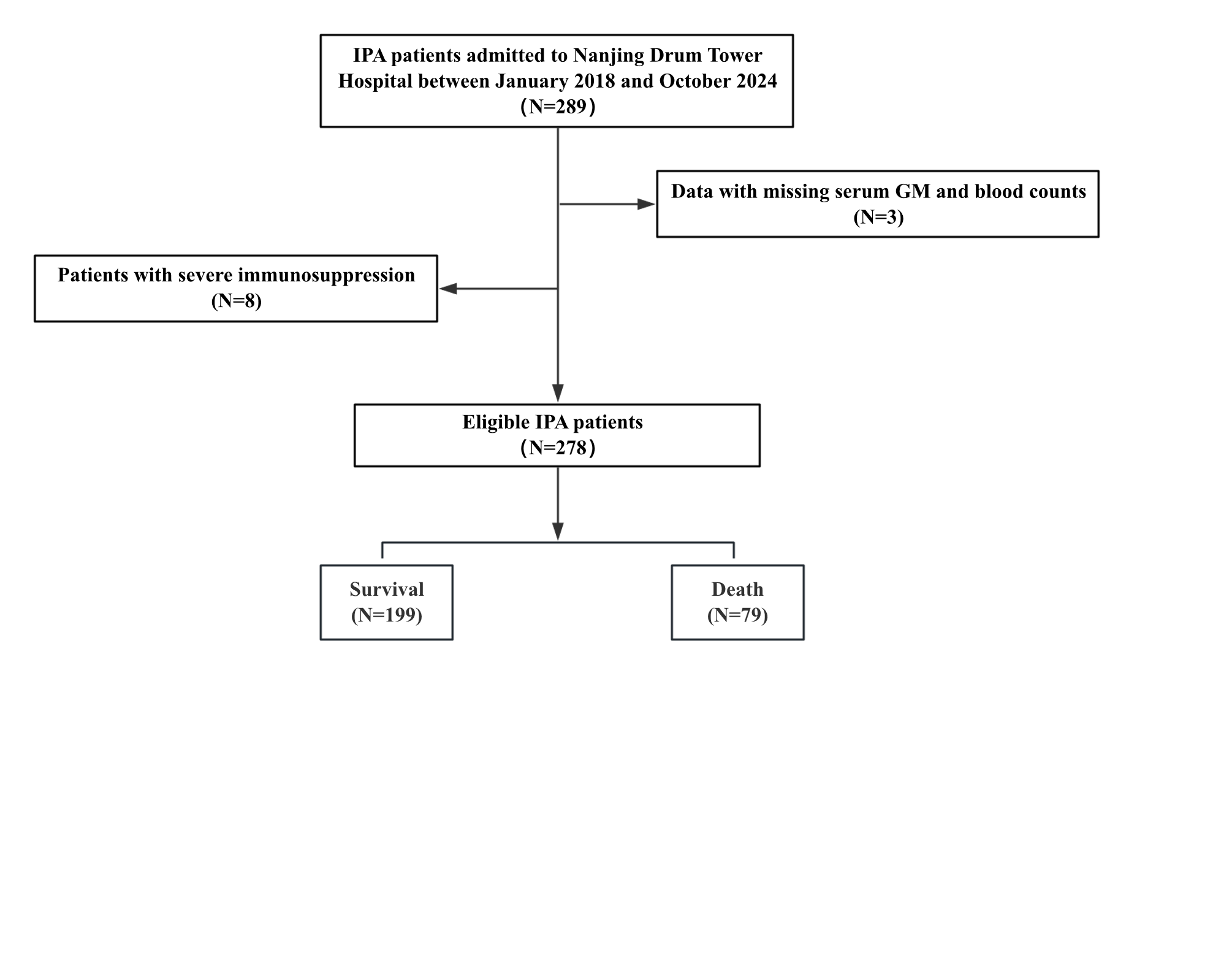


Figure legend: Flowchart of the study.

Note: severe immunosuppression: lymphocyte count of 0 or neutrophil count less than 0.5×10^9^/L.

Abbreviations: IPA: invasive pulmonary aspergillosis; GM: galactomannan.

# Supplementary Figure 2


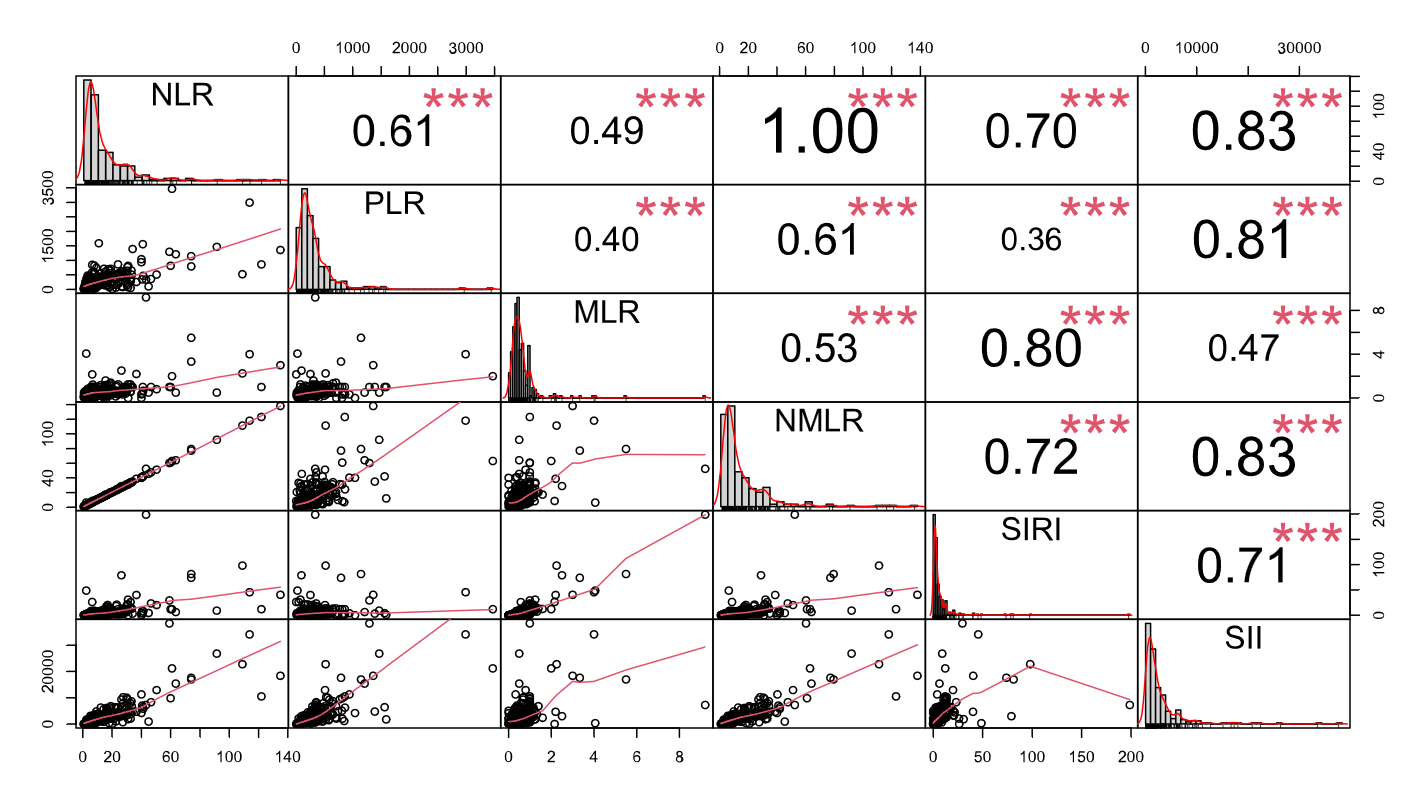


Figure legend: A correlation matrix among six variables: neutrophil-to-lymphocyte ratio (NLR), platelet-to-lymphocyte ratio (PLR), monocyte-to-lymphocyte ratio (MLR), neutrophil-monocyte-to-lymphocyte ratio (NMLR), systemic immune response index (SIRI), and systemic immune-inflammation index (SII). Values indicate Spearman correlation coefficients, with asterisks (***) denoting statistical significance levels (P < 0.001).

# Supplementary Figure 3


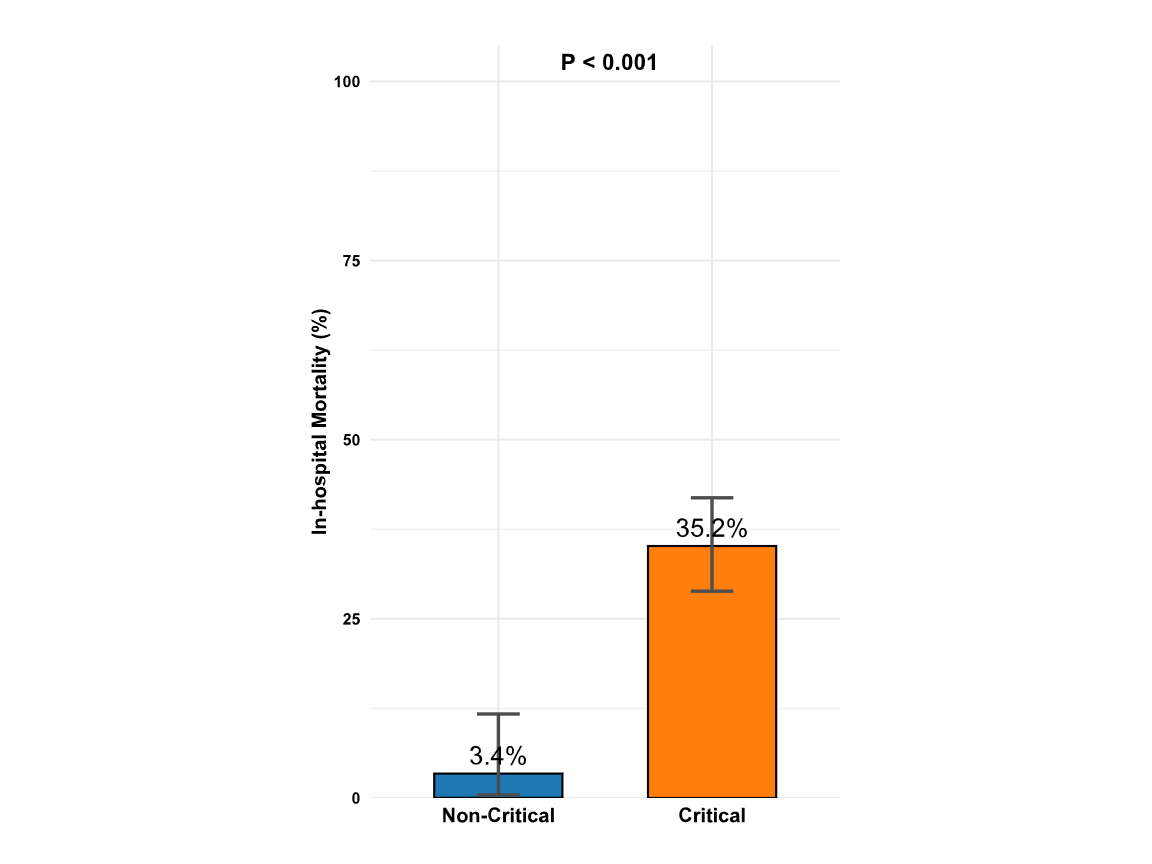


Figure legend: Comparison of in-hospital mortality between IPA patients in non-critical condition and critical condition. Error bars represent 95% confidence intervals. Intergroup comparisons revealed statistically significant differences (P < 0.001).

# Supplementary Figure 4


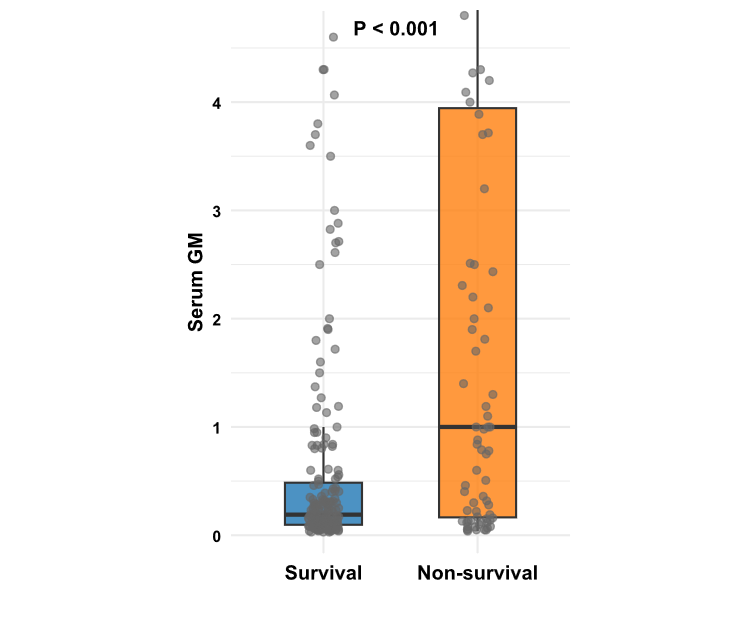


Figure legend: Distributions of serum GM in survival and non-survival groups

# Supplementary Figure 5


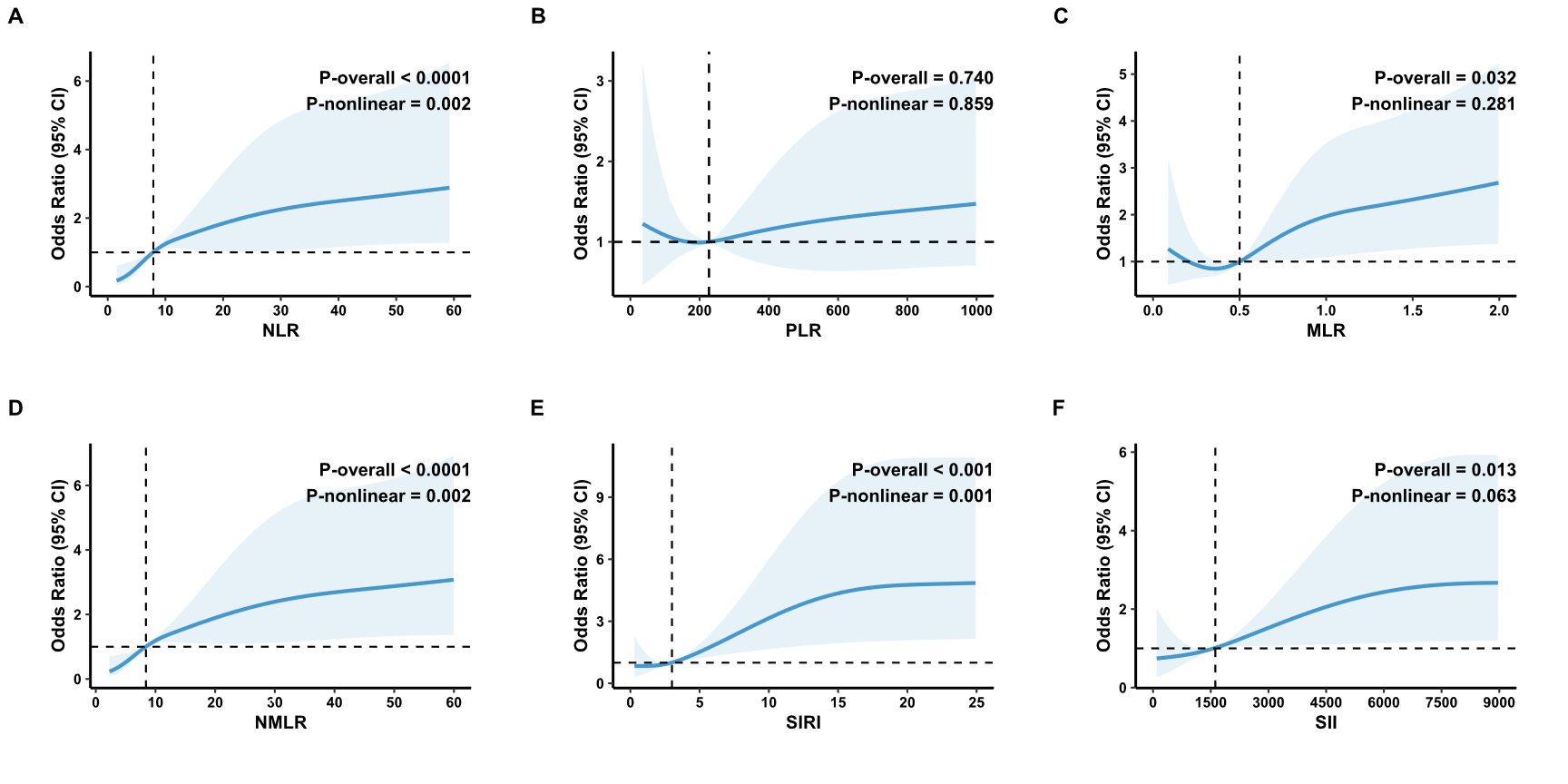


Figure legend: Restricted cubic splines of the association of NLR (A), PLR (B), MLR (C), NMLR (D), SIRI (E), and SII (F) with the odds of in-hospital mortality risk in IPA patients. The blue-shaded area indicates 95% confidence interval. Abbreviations: NLR: neutrophil-to-lymphocyte ratio; PLR: platelet-to-lymphocyte ratio; MLR: monocyte-to-lymphocyte ratio; NMLR: neutrophil-monocyte-to-lymphocyte ratio; SIRI: systemic inflammation response index; SII: systemic immune-inflammation index.

# Supplementary Figure 6


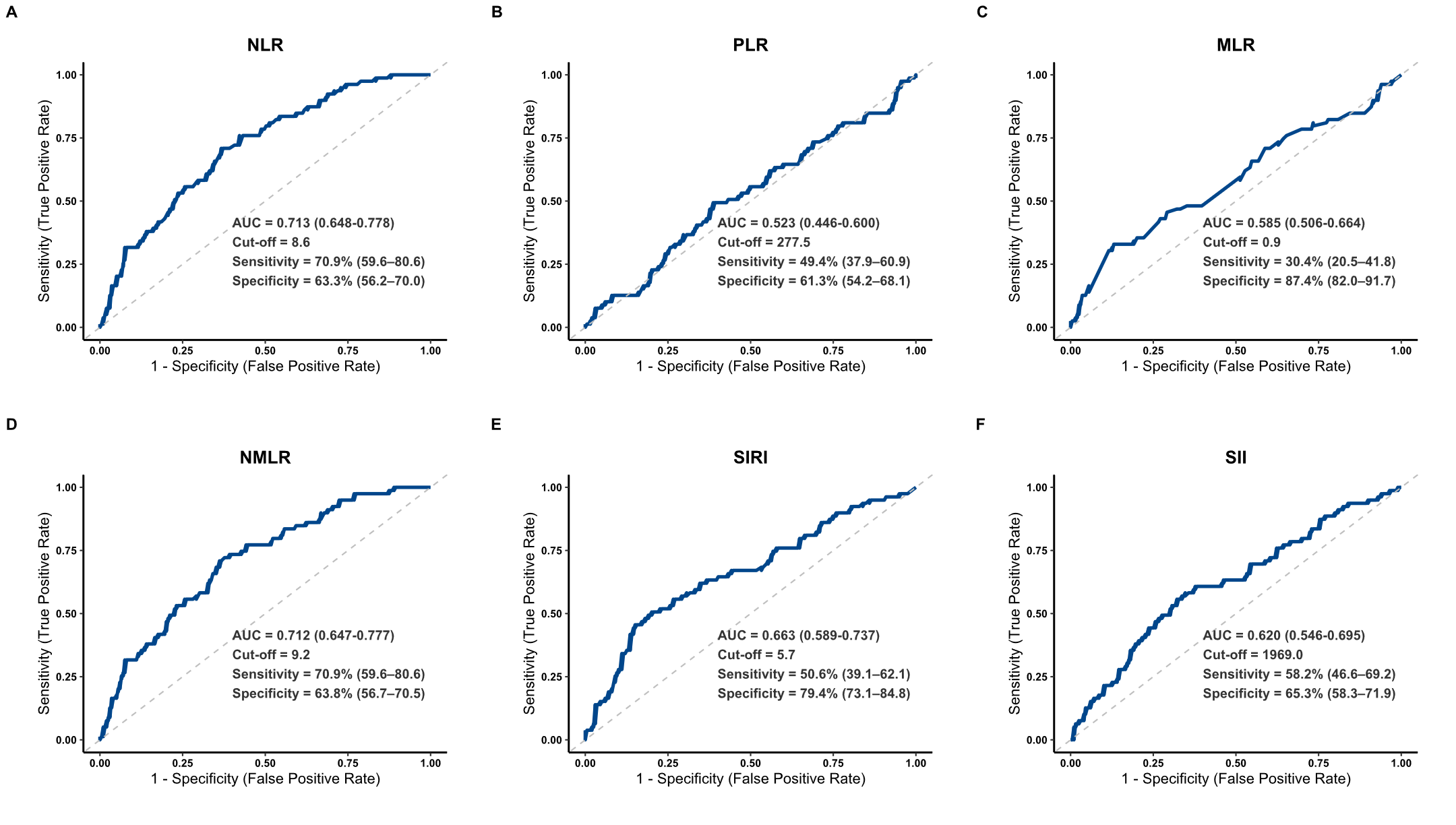


Figure legend: Receiver operator characteristic analyses for NLR (A), PLR (B), MLR (C), NMLR (D), SIRI (E), and SII (F) for predicting the in-hospital mortality in invasive pulmonary aspergillosis. Abbreviations: NLR: neutrophil-to-lymphocyte ratio; PLR: platelet-to-lymphocyte ratio; MLR: monocyte-to-lymphocyte ratio; NMLR: neutrophil-monocyte-to-lymphocyte ratio; SIRI: systemic inflammation response index; SII: systemic immune-inflammation index.

# Supplementary Figure 7


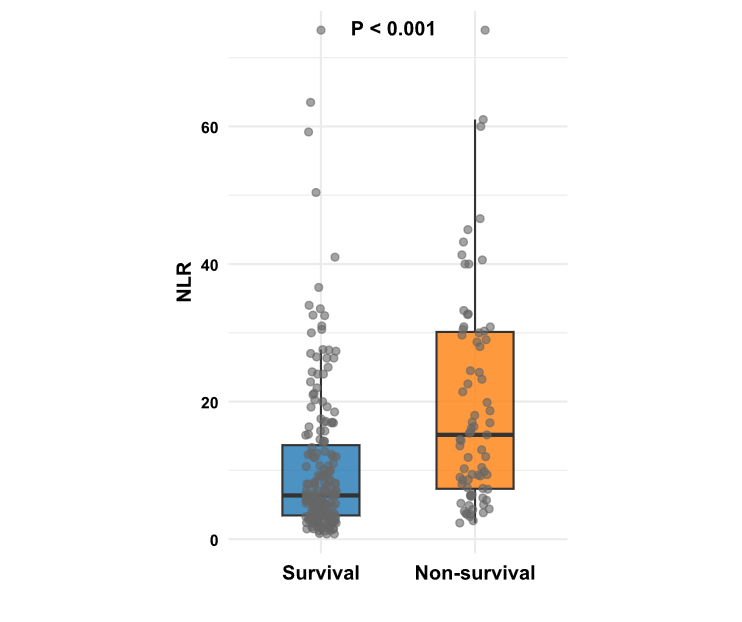


Figure legend: Distributions of NLR in survival and non-survival groups

# Supplementary Figure 8


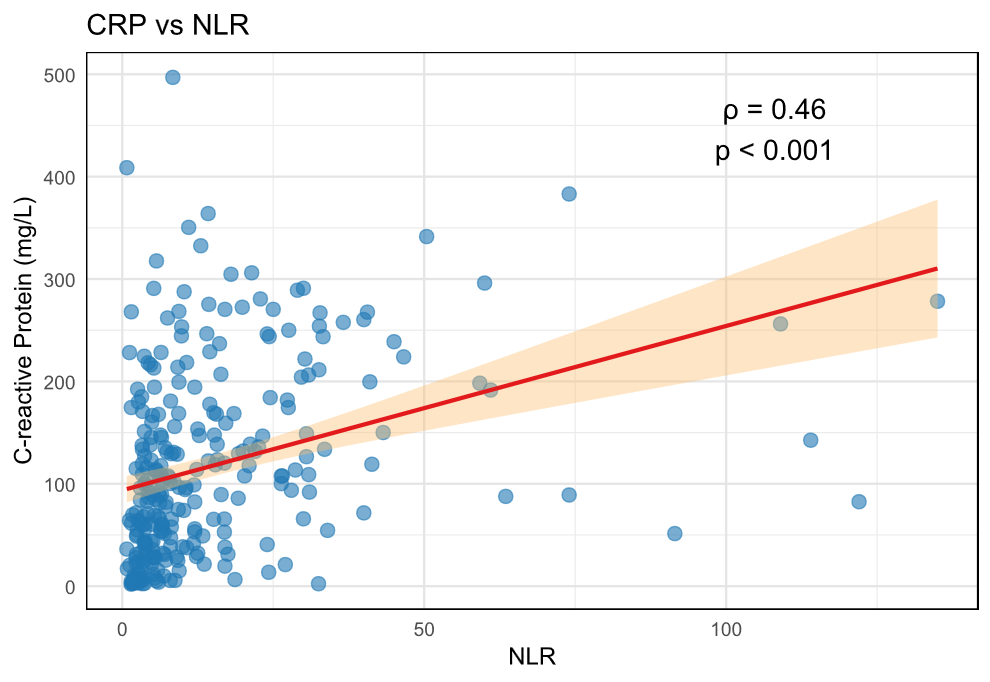


Figure legend: Correlation between neutrophil-to-lymphocyte ratio (NLR) and C-reactive protein. Values indicate Spearman correlation coefficients (ρ=0.46, P < 0.001).

# Supplementary Figure 9


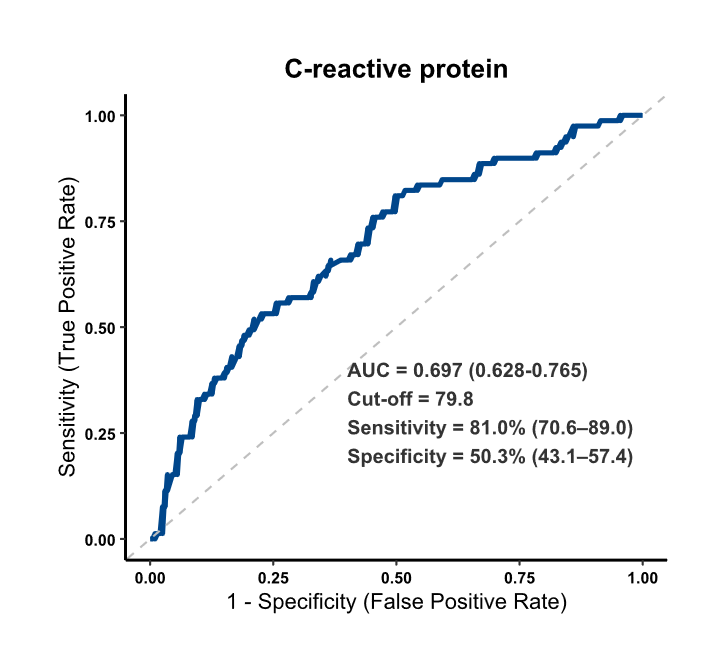


Figure legend: Receiver operator characteristic analyses for C-reactive protein level for predicting the in-hospital mortality in invasive pulmonary aspergillosis.

# Supplementary Figure 10


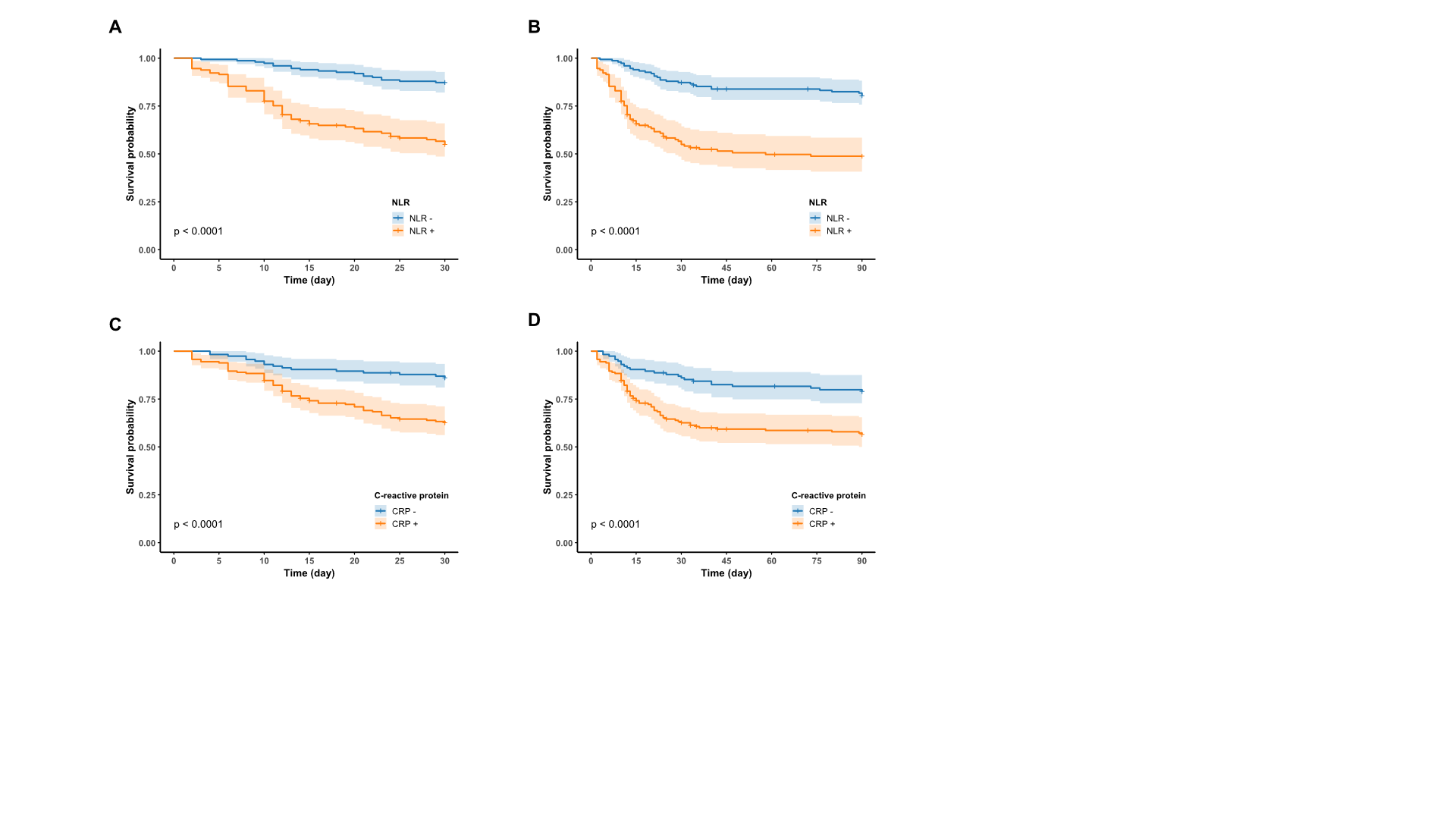


Figure legend: (A-B) Kaplan-Meier survival curve compares 30-day and 90-day survival between the NLR- and NLR+ group. (C-D) Kaplan-Meier survival curve compares 30-day and 90-day survival between the CRP- and CRP+ group. Note: NLR-: NLR < 8.6; NLR+: NLR ≥ 8.6; CRP +: C-reactive protein ≥ 79.8mg/L; CRP -< 79.8mg/L.

# Supplementary Figure 11


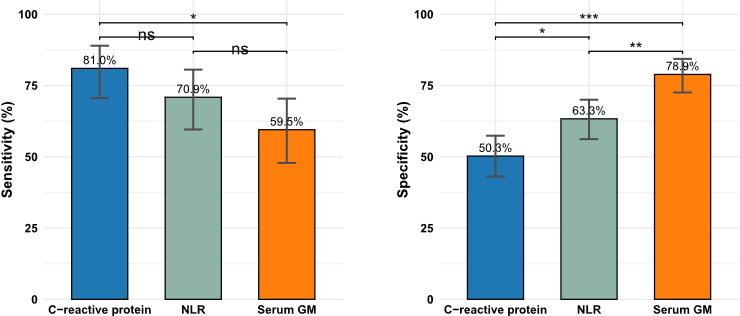


Figure legend. Comparison of the diagnostic performance between CRP (cut-off = 79.8 mg/L), NLR (cut-off = 8.6), and serum GM (cut-off = 0.7). CRP: C-reactive protein; NLR: neutrophil-to-lymphocyte ratio; GM: galactomannan.

# Supplementary Table 1

The co-infection and anti-*A*spergillus treatment among patients in survival and non-survival groups

|  | Survival (n=199) | Non-survival (n=79) | Chi-square | P-value |
| --- | --- | --- | --- | --- |
| Co-infection (%) | |  | 46.684 | <0.001 |
| None | 56 (28.1) | 3 (3.8) |  |  |
| Bacteria | 89 (44.7) | 48 (60.8) |  |  |
| Virus | 37 (18.6) | 5 (6.3) |  |  |
| Fungi (except *Aspergillus*) | 6 (3.0) | 1 (1.3) |  |  |
| Multiple | 12 (6.0) | 22 (27.8) |  |  |
| Anti-*Aspergillus* treatment (%) | | | 2.025 | 0.155 |
| Monotherapy | 157 (78.9) | 56 (70.9) |  |  |
| Combination therapy | 42 (21.1) | 23 (29.1) |  |  |

Note: "Multiple" refers to co-infections involving bacteria, viruses, or other fungi exclusive of Aspergillus. Patients with only one additional category of pathogen are not included in the “Multiple” groups.

# Supplementary Table 2

Cross-tab of BALF GM and serum GM positivity versus in-hospital mortality

|  |  | Survival | Non-survival |
| --- | --- | --- | --- |
| Serum GM- | BALF GM- | 45 | 6 |
| Serum GM+ | BALF GM- | 7 | 2 |
| Serum GM- | BALF GM+ | 73 | 23 |
| Serum GM+ | BALF GM+ | 12 | 25 |

Serum GM+: Serum GM ≥ 1.0; Serum GM-: Serum GM < 1.0;

BALF GM+: BALF GM ≥ 1.0; BALF GM-: BALF GM < 1.0;

Abbreviations: GM: galactomannan; BALF: bronchoalveolar lavage fluid

# Supplementary Table 3

Univariate logistic regression results for baseline variables and in-hospital mortality

|  | β | OR (95% CI) | P-value |
| --- | --- | --- | --- |
| Age (per 10 years) | 0.298 | 1.347 (1.076-1.713) | 0.012 |
| Critical Condition | 2.738 | 15.454 (4.643-95.905) | <0.001 |

# Supplementary Table 4

Confusion matrix of serum GM (cut-off = 0.7) for in-hospital mortality

|  | Non-survival | Survival |
| --- | --- | --- |
| Serum GM ≥ 0.7 | 47 | 42 |
| Serum GM < 0.7 | 32 | 157 |

# Supplementary Table 5

Sensitivity and specificity of serum GM for predicting in-hospital mortality at different thresholds

| Threshold | TP | FP | TN | FN | Sensitivity | Specificity |
| --- | --- | --- | --- | --- | --- | --- |
| 0.7 | 47 | 42 | 157 | 32 | 59.5% (47.9-70.4) | 78.9% (72.6-84.3) |
| 0.5 | 49 | 50 | 149 | 30 | 62.0% (50.4-72.7) | 74.9% (68.3-80.7) |
| 1.0 | 41 | 31 | 168 | 38 | 51.9% (40.4-63.3) | 84.4% (78.6-89.2) |

# Supplementary Table 6

Cox regression analysis of serum GM levels for 30-day and 90-day mortality

|  | HR (95% CI) | P-value |
| --- | --- | --- |
| 30-day mortality | |  |
| Serum GM | 1.375 (1.256-1.505) | <0.001 |
| Serum GM ≥ 0.7 | 3.093 (1.968-4.863) | <0.001 |
| 90-day mortality | |  |
| Serum GM | 1.350 (1.238-1.472) | <0.001 |
| Serum GM ≥ 0.7 | 2.829 (1.881-4.254) | <0.001 |

# Supplementary Table 7

Diagnostic performance and parameters of the CBC-derived inflammatory biomarkers

|  | NLR | PLR | MLR | NMLR | SIRI | SII |
| --- | --- | --- | --- | --- | --- | --- |
| AUC | 0.713 (0.648-0.778) | 0.523 (0.446-0.600) | 0.585 (0.506-0.664) | 0.712 (0.647-0.777) | 0.663 (0.589-0.737) | 0.620 (0.546-0.695) |
| Cut-off | 8.6 | 277.5 | 0.9 | 9.2 | 5.7 | 1969.0 |
| Sensitivity | 70.9% (59.6–80.6) | 49.4% (37.9–60.9) | 30.4% (20.5–41.8) | 70.9% (59.6–80.6) | 50.6% (39.1–62.1) | 58.2% (46.6–69.2) |
| Specificity | 63.3% (56.2–70.0) | 61.3% (54.2–68.1) | 87.4% (82.0–91.7) | 63.8% (56.7–70.5) | 79.4% (73.1–84.8) | 65.3% (58.3–71.9) |
| Positive predictive value | 43.4% (34.7–52.4) | 33.6% (25.1–43.0) | 49.0% (34.4–63.7) | 43.8% (35.0–52.8) | 49.4% (38.1–60.7) | 40.0% (31.0–49.6) |
| Negative predictive value | 84.6% (77.7–90.0) | 75.3% (67.9–81.7) | 76.0% (69.9–81.4) | 84.7% (77.9–90.0) | 80.2% (73.9–85.5) | 79.8% (72.8–85.6) |
| Accuracy | 65.5% (59.6–71.0) | 57.9% (51.9–63.8) | 71.2% (65.5–76.5) | 65.8% (59.9–71.4) | 71.2% (65.5–76.5) | 63.3% (57.3–69.0) |
| Youden index | 34.2% (15.8–50.6) | 10.7% (-7.9–29.0) | 17.8% (2.5–33.5) | 34.7% (16.3–51.1) | 30.0% (12.2–46.9) | 23.6% (4.9–41.1) |

Abbreviations: NLR: neutrophil-to-lymphocyte ratio; PLR: platelet-to-lymphocyte ratio; MLR: monocyte-to-lymphocyte ratio; NMLR: neutrophil-monocyte-to-lymphocyte ratio; SIRI: systemic inflammation response index; SII: systemic immune-inflammation index.

# Supplementary Table 8

Multivariable logistic regression analysis of GM/NLR groups for in-hospital mortality

| Variable | Coefficient | OR (95% CI) | P-value |
| --- | --- | --- | --- |
| Serum GM/NLR group | |  |  |
| GM- & NLR- | ref | ref | ref |
| GM+ / NLR+ | 0.635 | 1.887 (0.895-4.088) | 0.100 |
| GM+ & NLR+ | 2.509 | 12.289 (5.308-30.418) | <0.001 |
| Age | 0.045 | 1.046 (1.018-1.076) | 0.001 |
| Critical condition | 1.801 | 6.056 (1.624-39.865) | 0.021 |
| Combination therapy | 0.178 | 1.195 (0.567-2.483) | 0.635 |
| Co-infection | 2.042 | 7.702 (2.348-36.039) | 0.003 |

Serum GM+: Serum GM ≥ 0.7; Serum GM-: Serum GM < 0.7;

NLR+: NLR ≥ 8.6; NLR-: NLR < 8.6;

# Supplementary Table 9

In-hospital mortality across serum GM/NLR subgroups stratified by critical condition, ICU admission, and COPD status

|  | In-hospital mortality (%) | | | P-value |
| --- | --- | --- | --- | --- |
|  | serum GM- & NLR- | serum GM+ / NLR+ | serum GM+ & NLR+ |  |
| Critical condition | 15/79 (19.0) | 24/84 (28.6) | 38/56 (67.9) | <0.001 |
| Non-critical condition | 0/39 (0.0) | 1/18 (5.6) | 1/2 (50.0) | 0.022 |
| ICU admission | 10/31 (32.3) | 17/47 (36.2) | 33/40 (82.5) | <0.001 |
| Non-ICU admission | 5/87 (5.7) | 8/55 (14.5) | 6/18 (33.3) | 0.003 |
| COPD | 3/33 (9.1) | 6/24 (25.0) | 7/9 (77.8) | <0.001 |
| Non-COPD | 12/85 (14.1) | 19/78 (24.4) | 32/49 (65.3) | <0.001 |

Serum GM+: Serum GM ≥ 0.7; Serum GM-: Serum GM < 0.7;

NLR+: NLR ≥ 8.6; NLR-: NLR < 8.6;

The differences between groups were assessed using Chi-square or Fisher’s exact test.

# Supplementary Table 10

Multivariable logistic regression model for in-hospital mortality

| Variable | β | OR (95% CI) | P-value |
| --- | --- | --- | --- |
| Age | 0.037 | 1.038 (1.012-1.065) | 0.004 |
| Critical condition | 2.283 | 9.811 (2.221-43.343) | 0.003 |
| Serum GM ≥ 0.7 | 1.569 | 4.802 (2.552-9.032) | <0.001 |
| NLR ≥ 8.6 | 0.962 | 2.617 (1.393-4.916) | 0.003 |

# Supplementary Table 11

Net benefit of the model and simple model at threshold probabilities from 0.1 to 0.9

| Threshold | Simple model | Model |
| --- | --- | --- |
| 0.1 | 0.220 | 0.223 |
| 0.2 | 0.141 | 0.170 |
| 0.3 | 0.065 | 0.128 |
| 0.4 | 0.043 | 0.073 |
| 0.5 | 0.000 | 0.065 |
| 0.6 | 0.000 | 0.016 |
| 0.7 | 0.000 | 0.041 |
| 0.8 | 0.000 | 0.000 |
| 0.9 | 0.000 | 0.000 |
